# Development of a computer-aided design and finite-element analysis combined method for customized Nuss bar in pectus excavatum surgery

**DOI:** 10.1038/s41598-017-03622-y

**Published:** 2017-06-14

**Authors:** Longhan Xie, Siqi Cai, Liang Xie, Gang Chen, Haiyu Zhou

**Affiliations:** 1 School of Mechanical and Automotive Engineering, South China University of Technology, Guangzhou, 510640 China; 2 Department of Thoracic Surgery, Guangdong General Hospital & Guangdong Academy of Medical Sciences, Guangzhou, 510080 China; 3 School of Medicine, South China University of Technology, Guangzhou, 510640 China

## Abstract

Pectus excavatum (PEX) is the most common chest deformity in children, which is usually corrected by using the minimally invasive Nuss method. The orthopedic effect of the Nuss operation is mainly evaluated by both the Haller index and the appearance of the chest wall configuration, which is dependent on the operator’s clinical experience to a great extent. To improve the orthopedic effect, we proposed a novel method to individually design and optimize the shape of the Nuss bar and to advise its location as well as the incisions. By using the CT imaging data, the three-dimensional model of the PEX thoracic structure is reconstructed, which is further employed in finite element analysis to determine the operation plan. By referring to a healthy person who has similar chest dimensions to the PEX patient, the Nuss bar shape is designed, and according to the finding that the healthy chest wall boundary is almost convex with positive curvature, the Nuss bar shape is tuned to be a convex curve to ensure the orthopedic effect. Finite element analysis is employed to analyze the orthopedic effect and to determine the incision position of the Nuss bar. Experiments were carried out to verify the orthopedic effect of the customized Nuss bar, which showed that this method is more accurate and individualized, compared to conventional methods.

## Introduction

Pectus excavatum (PEX) is the most common chest deformity in children, which is characterized by a depressed sternal region relative to the frontal rib cage^1^. The deformity decreases the volume of the chest and affects the circulatory and respiratory systems, and it even has a negative impact on the patient’s mental health^2^.

In 1998, a minimally invasive repair of pectus excavatum was reported by Nuss *et al*.^3^. This procedure involves inserting a convex Nuss bar under the sternum through small bilateral thoracic incisions without incising the anterior chest wall^4^. Because of small skin incisions, shorter preoperative, intraoperative and post-operative time, and minimal blood loss, this method is accepted by an increasing number of patients with pectus excavatum^5–7^. Although the Nuss procedure is routinely performed, the outcome depends mostly on the correct placement of the bar^8^. From the mechanic view, the sternum is a beam with displacement constraint from ribs, so when the beam is elevated with a distance at one point, ribs would constrain the elevation of the sternum by elastic deformation, which could complicatedly influence the deformation of the thoracic structure. And thus, the interaction forces among sternum and ribs should be taken into consideration during determining the placement position of the Nuss bar. Since the deformation of thoracic structure is very complicated, a Nuss procedure surgical planner would be an invaluable planning tool ensuring the optimal aesthetic outcome^9^. This means that the location of the incisions and the shape of the Nuss bar are determined only by the physician with clinical experience. Sometimes the physician needs to adjust the shape of the Nuss bar many times during operation, especially when the depression of the patients’ chest is complicated, which may lead to prolonged the operation time, increased blood loss and the possibility of damage to the patient. To determine the shape of the Nuss bar before operation, some researchers have developed a non-invasive procedure to reconstruct the thorax and construct the shape of the Nuss bar by referring to healthy subjects^10–14^; although these methods can obtain the desired shape of the Nuss bar, the chest wall deformity and stress distribution after Nuss bar implantation was not investigated. Actually, Nuss bar has a high risk of failure due to poor stress distribution in pectus excavatum. Quan Li drew the conclusion that stainless steel instruments introduce higher risk for rod failure and are less favorable for lumboiliac arthrodesis than titanium instruments^15^. Mingyi Wang gave advice on restoring unilateral maxilla defects after simulating the defects in the maxillofacial model and reconstructing the model with different methods^16^, where the finite element analysis to optimize prostheses surgery can be a good reference for the Nuss operation. Thus, both the shape of the Nuss bar and the stress distribution of the chest after implantation are of equal importance.

In this study, we proposed a novel method to individually design and optimize the shape of the Nuss bar and to advise its location of the incisions using CT imaging data of the patient’s thorax. In the proposed method, a three-dimensional model of the human thorax is established first using the data obtained from a CT scan of a patient with a funnel chest. Then, we construct the finite element model, in which different operative plans are simulated. The optimum plan is chosen according to the chest wall configuration and the stress distribution. Then, we compare it with a normal person whose chest wall configuration is similar to the patient and adjust the shape of the Nuss bar. Finally, experiments using customized Nuss bars were carried out to validate the method in this paper.

## Methods

### 3D Reconstruction of Thoracic Structure

**Figure 1:**
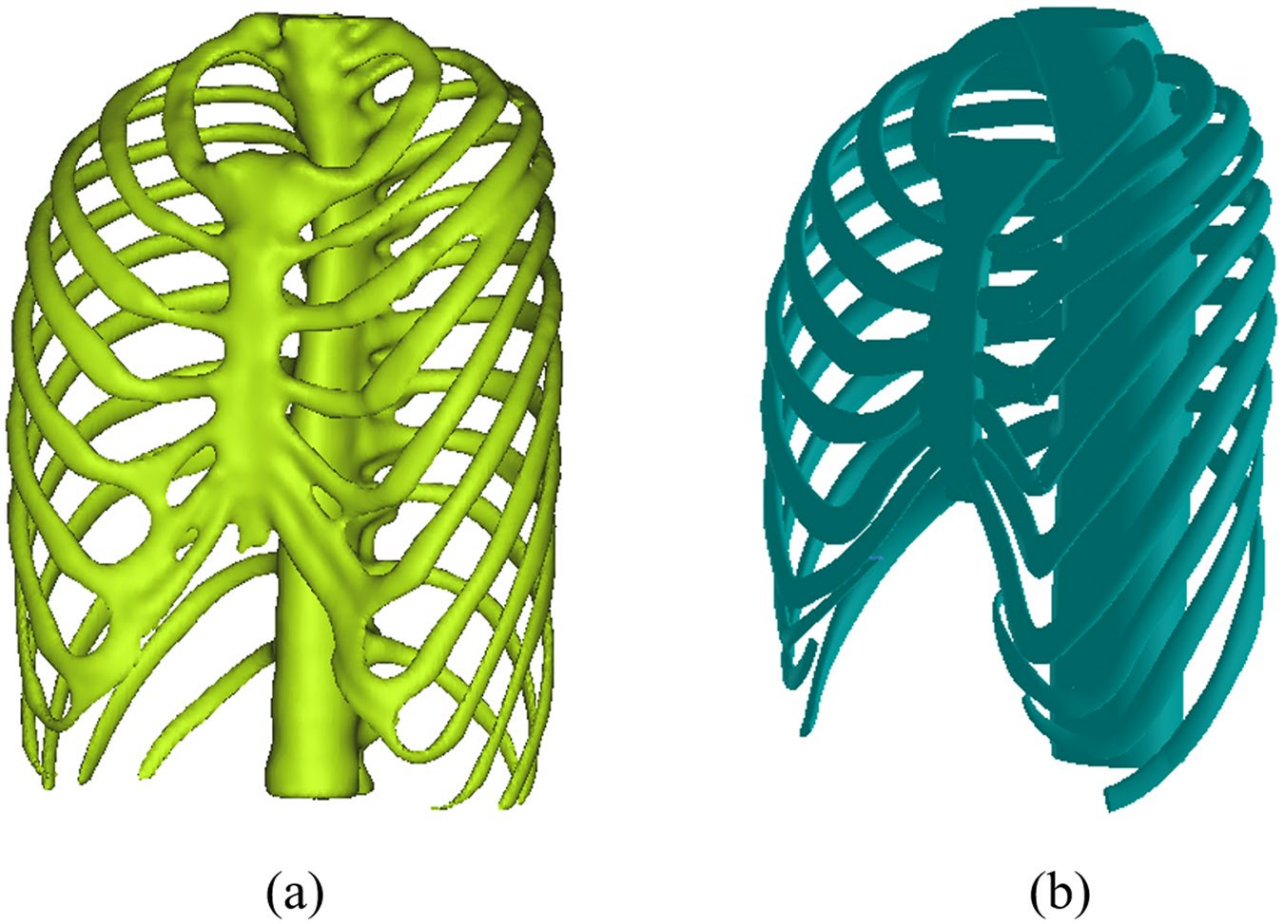
(**a**) The 3D model of the thorax constructed with DICOM data. (**b**) The 3D model of the thorax reconstructed by SolidWorks software.

The shape design and analysis of the Nuss bar are based on the thoracic structure of PEX patients, thus the first step to reconstruct the three-dimensional model of the thoracic structure is to use imaging data from a CT scan. The chest of the PEX patient is scanned by a clinical CT scanner with a slice thickness of 0.625 mm, and the imaging data is saved as Digital Imagine and Communications in Medicine (DICOM) files. Then, the DICOM data is imported into the MIMICS^®^ software (Materialise, Belgium) which is good at reconstructing accurate 3D models from CT imaging data. This procedure has served to separate the thoracic structure from the rest of the chest structures on each image, such as the shoulder blades. Based on the analysis of the pixel intensity curve of the CT image, threshold selection can remove redundant data and create a new mask by region growing. Based on this mask, the three-dimensional model can be reconstructed by calculation. Figure 1(a) gives an example of a reconstructed 3D model of the thoracic structure.

In addition to designing the bar shape, the thoracic structure is also used to determine the location of the incisions and to analyze the stress by finite element method. By using imaging examination, it can be observed that ribs, cartilage and sternum vary significantly while spine has only slightly change after Nuss operation. In order to reduce the amount of calculation, the spine is treated as a rigid object and simplified as a cylinder in finite element analysis. With the simplified thoracic structure, ribs are also reconstructed in solid models to decrease the computational complexity in finite element analysis. Figure 1(b) shows the simplified thoracic structure model reconstructed in SolidWorks^®^ software.

### Shape Design of Nuss Bar by Referring to a Similar Healthy Chest

**Figure 2:**
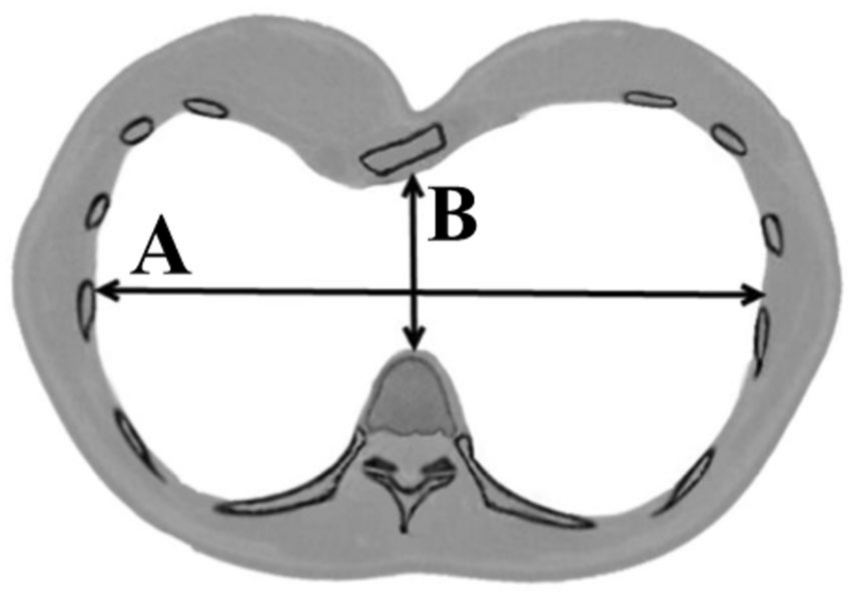
Schematic calculation of the Haller index = A/B.

The Haller index is usually used to assess the severity of chest wall deformities, which is convenient to measure and calculate. The Haller index is based on computed tomography of the chest and is defined as the quotient between the maximum laterolateral distance and the minimum anteroposterior distance from the anterior portion of the vertebral body to the posterior surface of the sternum, as shown in Fig. 2 ^17^. From the formula for the Haller index H = A/B, it can be noted that there are two indices that affect the index value, that is, the thoracic transverse diameter A and the anterior-posterior diameter B. Therefore, both of these two influence factors should be taken into consideration during the shape design of Nuss bars.

The severest depression of the patient’s chest wall is determined by the model reconstructed by the MIMICS^®^ software. The pre- and post-operative thoracic transverse diameter A and $A^{\prime}$ of twelve patients with funnel chests, as well as the ratio of A and $A^{\prime} ,$ are presented in Table 1. From data in the table, it can be evidently noted that thoracic transverse diameter decreases after the Nuss procedure, and the shrinkage rate (A′/A) is approximately 95%. As the shrinkage will affect the value of the Haller index, it must be taken into account in the Nuss bar design to avoid overcorrection. Therefore, when selecting a healthy person as a reference for Nuss bar design, the referenced person is determined according to both the thoracic transverse diameter and the shrinkage rate.

**Table 1:** The pre- and post-operative thoracic transverse diameter of PEX patients.

Patient	Preoperative A(mm)	Post-operative A′(mm)	A′/A
1	268.05	253.98	0.95
2	223.31	210.83	0.94
3	264.35	250.56	0.95
4	222.76	217.92	0.98
5	254.97	242.77	0.95
6	249.98	243.46	0.97
7	204.51	194.73	0.95
8	212.23	203.29	0.96
9	245.81	232.09	0.94
10	240.91	235.48	0.98
11	232.30	211.71	0.91
12	250.38	228.63	0.91

**Figure 3:**
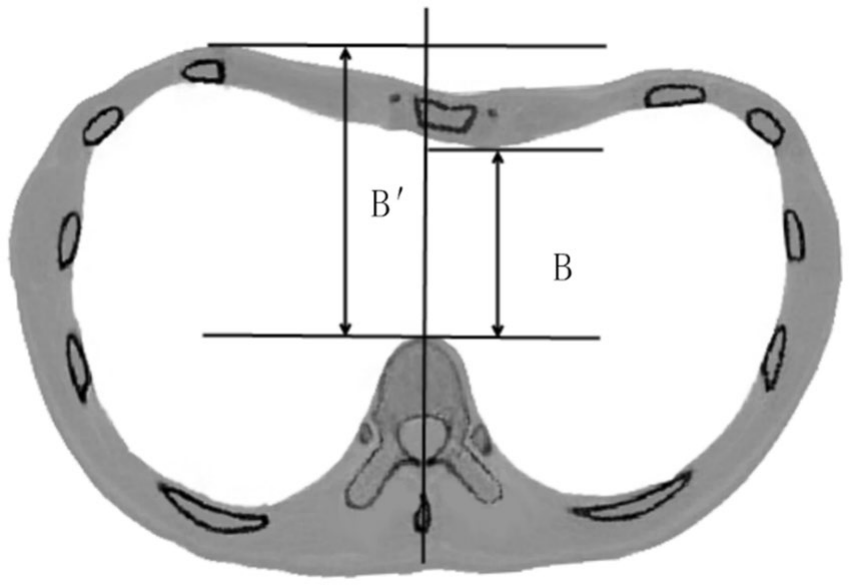
The pre- and post-operative anterior-posterior diameters.

Similar to the thoracic transverse diameter, the minimum anteroposterior distance from the anterior portion of the vertebral body to the posterior surface of the sternum is also changed after operation. As shown in Fig. 3, the minimum anteroposterior distance before operation is B, which is measured in the CT images, and the desired orthopedic minimum anteroposterior distance is marked as $B^{\prime}$, which could be determined using finite element analysis (FEA). In the FEA procedure, the Nuss bar is placed at different intercostal space and the sternum is elevated by different distances, and then the values of the orthopedic anteroposterior distance can be measured from the simulation results. From these values, the desired orthopedic minimum anteroposterior distance is evaluated by calculation of the Haller index. Let $\bigtriangleup =B^{\prime} -{\rm{B}}$ be the orthopedic distance of the PEX chest, which can be a design reference of the Nuss bar shape.

**Figure 4:**
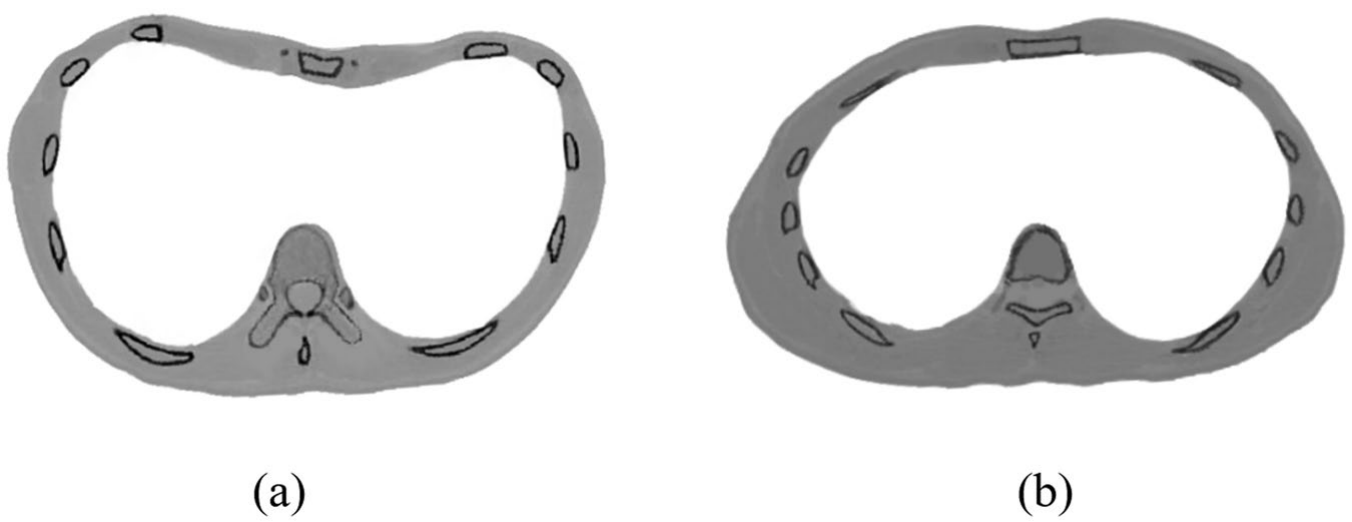
CT images of the chest. (**a**) The CT image of a PEX patient. **(b**) The CT image of the referenced healthy person.

The initial shape of the Nuss bar is designed by referring to a healthy person who has similar thoracic transverse diameter, age and gender to the PEX patient. For example, before designing the bar shape for a PEX patient, a referenced healthy person is selected from a CT imaging database. The cases in the CT imaging database, collecting CT scan data, are identified by age, gender, and thoracic transverse diameter. As shown in Fig. 4, the similar healthy person was selected to be a reference for designing the bar shape. Using the same method of reconstructing the PEX thoracic structure, the thoracic 3D model of the referenced healthy person is also reconstructed using the MIMICS software.

**Figure 5:**
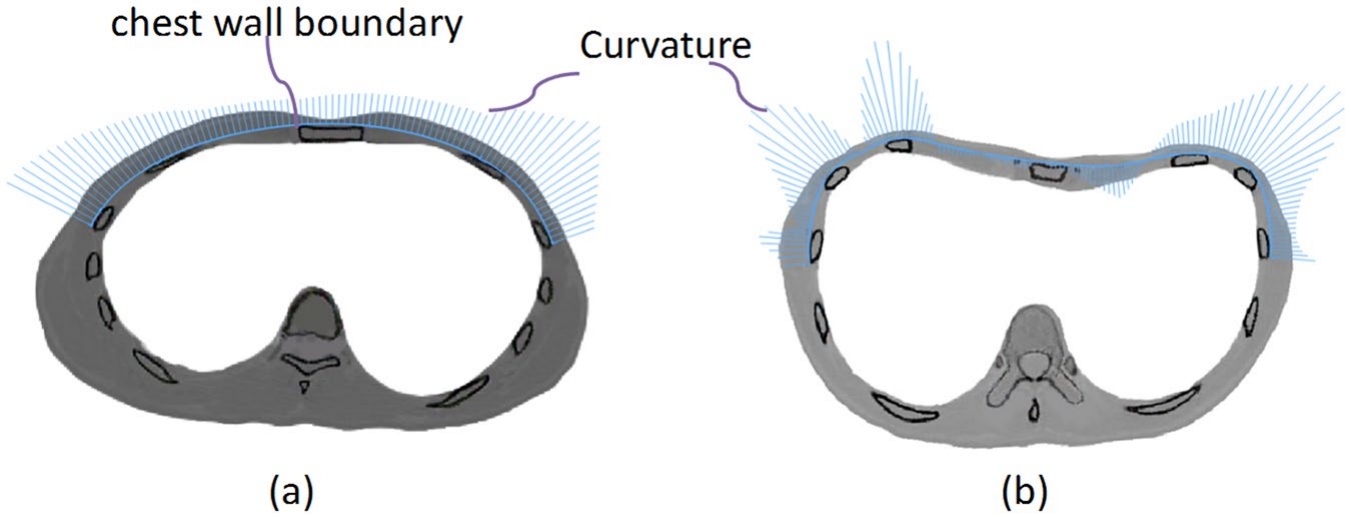
The chest wall boundary curves from the CT image. (**a**) The chest wall boundary curves of the patient A. (**b**) The chest wall boundary curves of the control in corresponding position.

**Figure 6:**
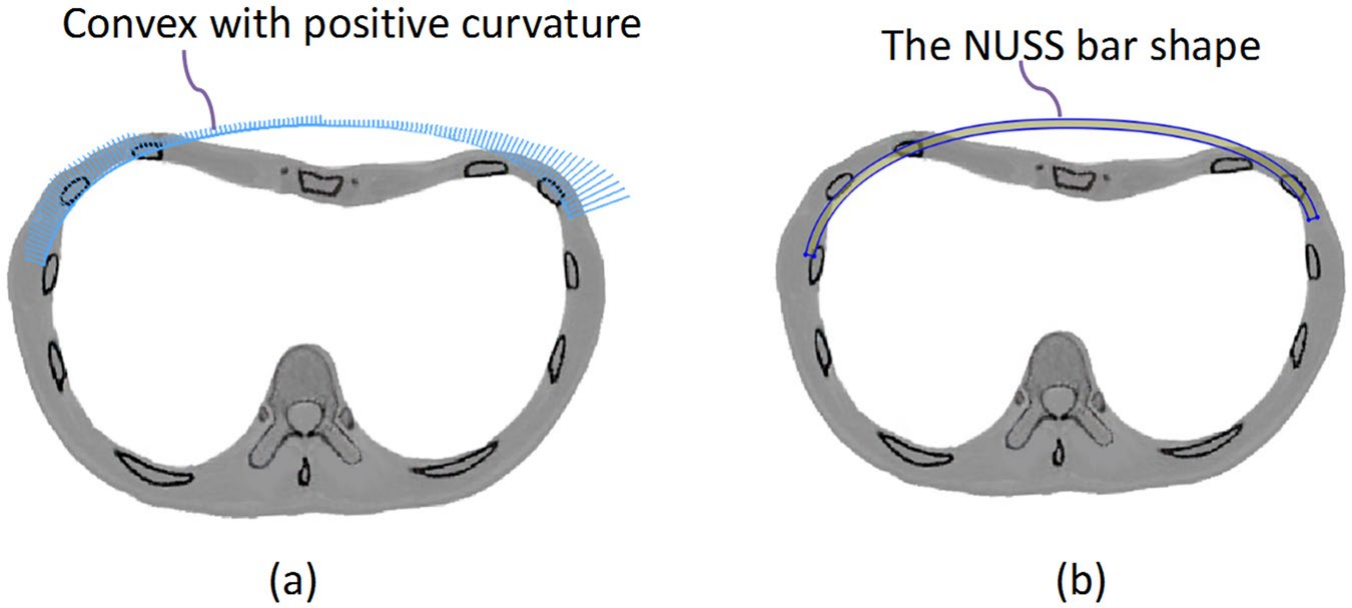
Design the spline curve of the Nuss bar. (**a**) The curve is convex with positive curvature. (**b**) The initial shape of the Nuss bar, which is also designed according to the operation request.

The curvature is positive when the curve is convex, and vice versa. Analyzing the curvature of the chest wall boundary of a healthy person, as shown in Fig. 5(a), it could be obviously noted that the chest wall boundary is almost convex with positive curvature. Taking the chest wall boundary of PEX patients into consideration, its curvature shows positive and negative variations in the patient’s chest wall boundary, as shown in Fig. 5(b). Thus, the Nuss bar shape should be designed as a convex curve to ensure the correction; Fig. 6 gives an example of a Nuss bar design, which has a convex curve. Considering that the Nuss bar used in clinical practice is an integral length and with one fixed end^18^, the Nuss bar is rounded off to a standard size.

### Evaluation of orthopedic operation by Finite Element Analysis

The initial shape and dimension of the Nuss bar is analyzed using finite element analysis to evaluate the orthopedic effect after implantation. With the 3D model built in SolidWorks^®^, ANSYS^®^ workbench is used to conduct the finite element analysis (FEA). All materials involved in the model are assumed to be isotropic, homogenous, linearly elastic and static. The spine and ribs are assumed to be cancellous bone^19^ to simplify the model and reduce calculation time. Thus, there are two kinds of materials in this model, cancellous bone and costal cartilage. The properties of materials used in this study, including Young’s modulus, Poisson’s ratio and density, obtained from the literature^20–23^, are listed in Table 2.

**Table 2:** Material properties for FEA.

Material	Density (kg/m^−3^)	Young’s modulus (Pa)	Poisson’s ratio
cancellous bone	1.67 × 10^5^	4.55 × 10^8^	0.3
costal cartilage	1.17 × 10^5^	3.75 × 10^7^	0.3

To make the finite element analysis possible, two boundary conditions are assumed in the analysis. First, considering that the rib, cartilage and sternum vary significantly while the spine does not obviously change in the Nuss operation, the spine is assumed to be fixed during the thorax correction simulation. Second, the elevation of the sternum is assumed to be vertical during operation. Based on these assumptions, the boundary condition of the spine is a fixed constraint and the loading direction of the sternum is vertical.

**Figure 7:**
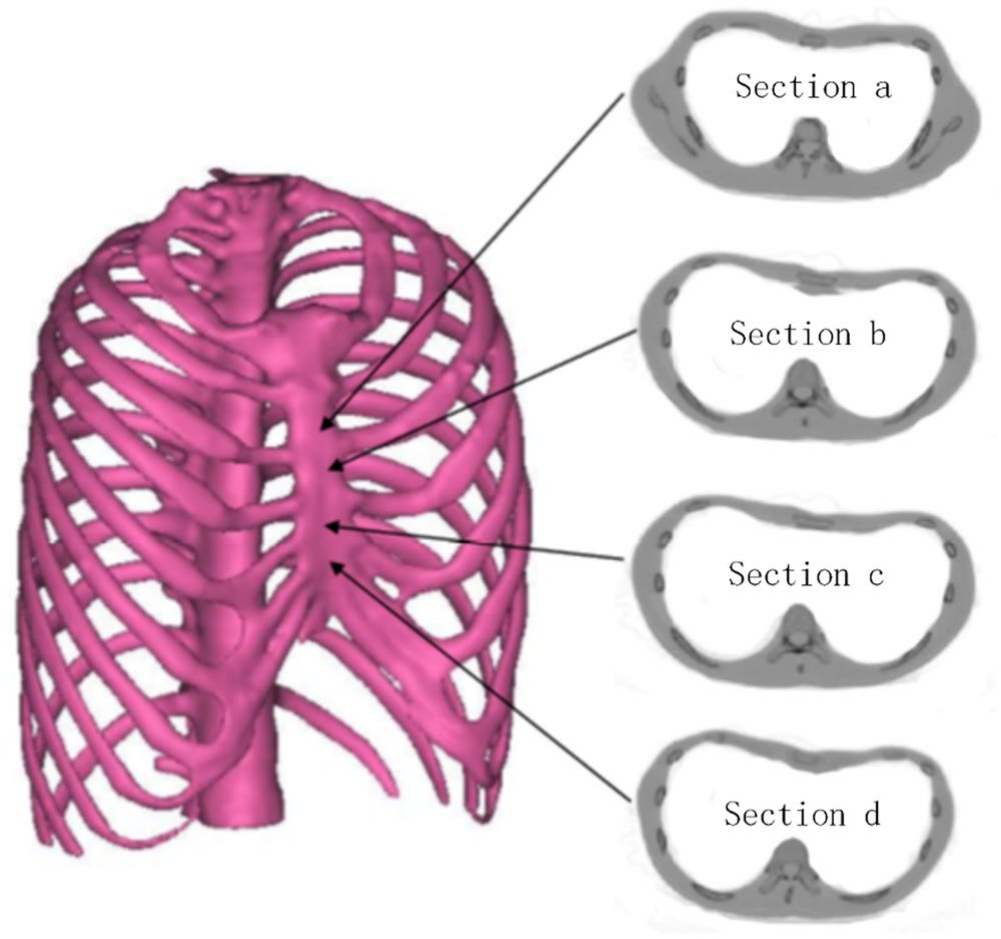
The Haller index is measured before and after correction in the 2nd, 3rd, 4th and 5th intercostal space.

To evaluate the efficacy of the surgical simulation in finite element analysis, the Haller index is measured before and after correction in the 2nd, 3rd, 4th and 5th intercostal space, as shown in Fig. 7. Then, we simulate different operative strategies, including placing the Nuss bar in the 2nd, 3rd, 4th and 5th intercostal space, respectively, and set different elevations in every intercostal space. The results of deformation and equivalent stress of each operative plan are recorded which is used as criteria for determining the optimal plan.

The study was done in accordance with the regulations of the National Health and Family Planning Commission of the People’s Republic of China. Guangdong General Hospital medical ethics committee approved this study and All study participants provided informed consent.

## Results

Because of its nature of being non-invasive and flexible to simulate different plans of operation for the same patient and to see the therapeutic effects of the patients clearly, finite element analysis using ANSYS is employed to assess the correction effect of the Nuss bar chest implantation.

**Figure 8:**
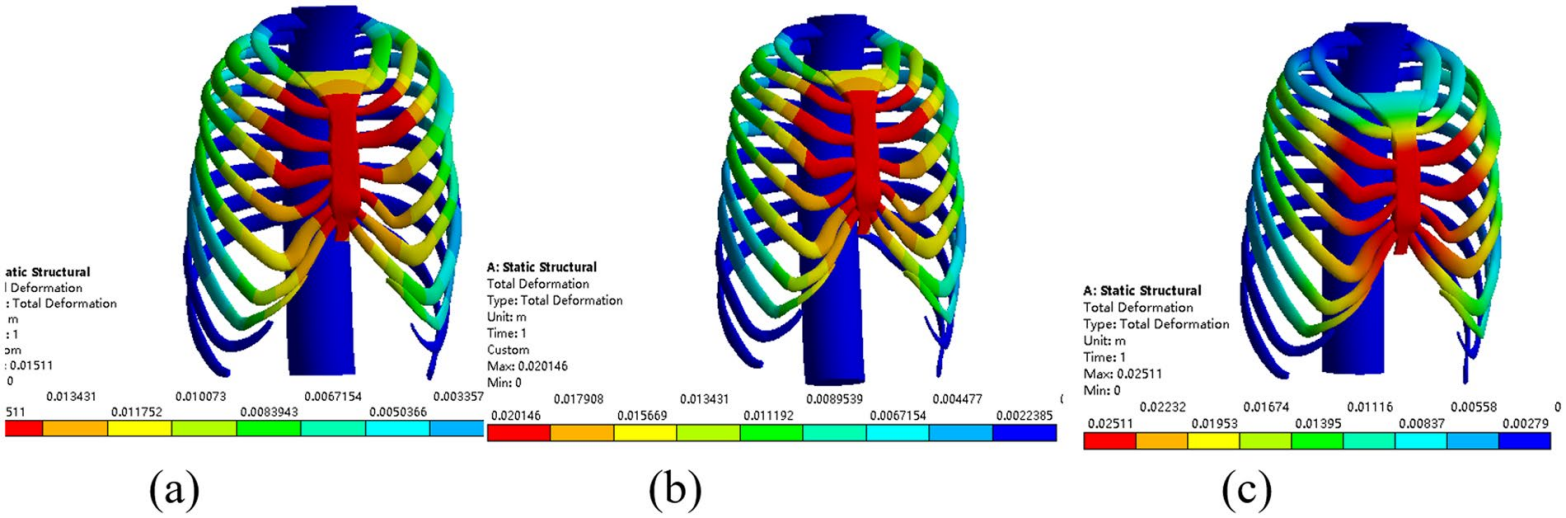
The correction effect of the chest model. (**a**) The sternum is elevated 15 mm at the 2nd intercostal space. (**b**) The sternum is elevated 20 mm at the 2nd intercostal space. (**c**) The sternum is elevated 25 mm at the 2nd intercostal space.

**Figure 9:**
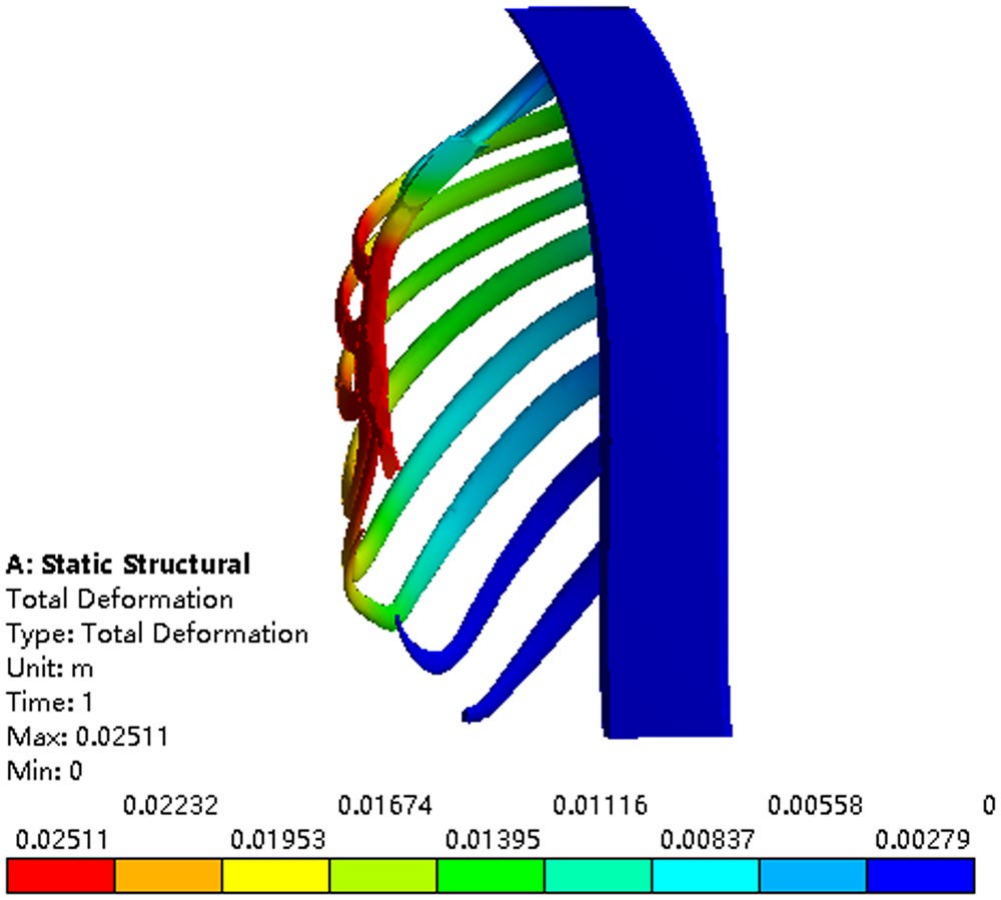
The sectional view of the sternum with 25 mm elevation at the 2nd intercostal space.

First, the Nuss bar is simulated to be placed at the 2nd intercostal space with the sternum elevated 15 mm, 20 mm and 25 mm, respectively, and the efficacy is compared, as shown in Fig. 8. The values of the Haller index in four cross sections before and after operation are given in Tables 3~6. According to the conclusion drawn by Lin^24^, who analyzed 252 patients with funnel chest pre- and post-operative Haller index, their mean value of post-operative Haller index of 2.68 was used as the criterion of corrective Haller index in this study. Therefore, the operative plans of elevating 15 mm and 20 mm in the 2nd intercostal space cannot meet the demand. When the elevation distance is 25 mm, the post-operative Haller index barely meets the requirement. Nevertheless, this plan causes hypercorrection of the upper end of the sternum, which can be obviously seen in the sectional view, as shown in Fig. 9. The overcorrection not only leads to unreasonable appearance but also results in stress concentration in the sternum. All of the analysis shows that putting the Nuss bar in the 2nd intercostal space is not a suitable scheme for the patient.

**Table 3:** The Haller index of patient A before the operation.

Cross-section	Distance B	Distance A	The Haller index
a	72.10	246.23	3.42
b	74.01	251.64	3.40
c	74.76	254.72	3.41
d	68.18	253.98	3.73

**Table 4:** The Haller index of patient A after the operation by placing the Nuss bar at the 2nd intercostal space with the sternum elevated 15 mm.

Cross-section	Distance B	Distance A	The Haller index
a	87.21	234.23	2.686
b	89.12	241.47	2.709
c	89.87	248.73	2.768
d	83.29	250.68	3.010

**Table 5:** The Haller index of patient A after the operation by placing the Nuss bar at the 2nd intercostal space with the sternum elevated 20 mm.

Cross-section	Distance B	Distance A	The Haller index
a	92.246	229.28	2.486
b	94.156	238.34	2.531
c	94.906	245.29	2.585
d	88.326	248.78	2.817

**Table 6:** The Haller index of patient A after the operation by placing the Nuss bar at the 2nd intercostal space with the sternum elevated 25 mm.

Cross-section	Distance B	Distance A	The Haller index
a	97.21	225.98	2.325
b	99.12	234.59	2.367
c	99.87	243.28	2.436
d	93.29	245.92	2.636

**Figure 10:**
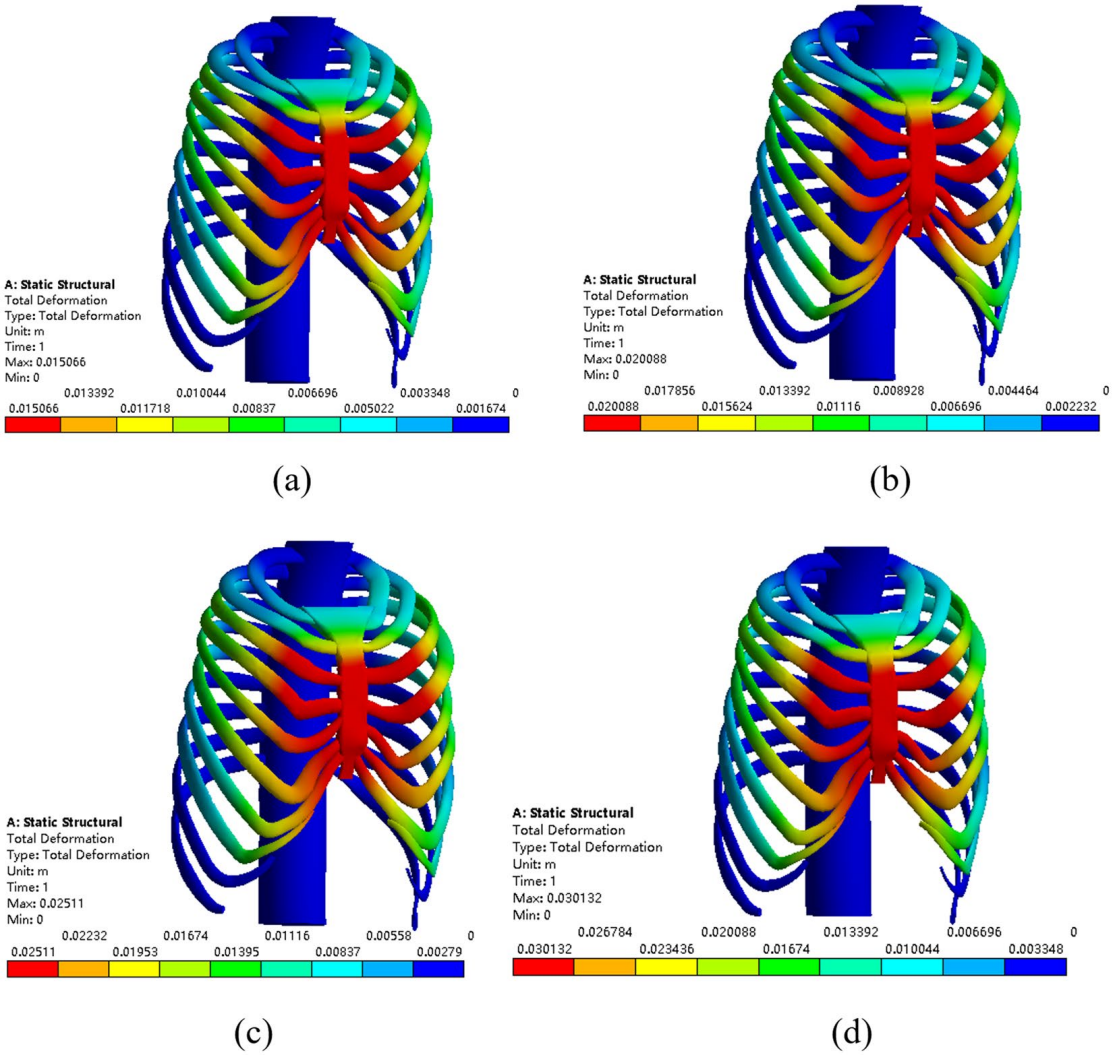
The correction effect of the chest model. (**a**) The sternum is elevated 15 mm at the 3rd intercostal space. (**b**) The sternum is elevated 20 mm at the 3rd intercostal space. (**c**) The sternum is elevated 25 mm at the 3rd intercostal space. (**d**) The sternum is elevated 30 mm at the 3rd intercostal space.

**Figure 11:**
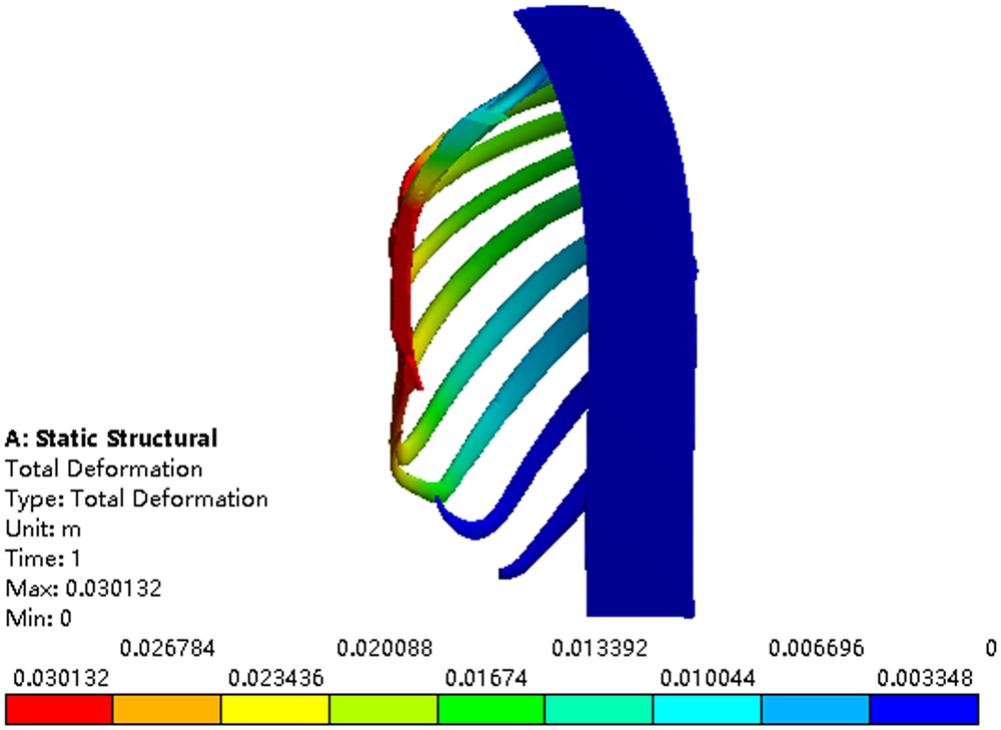
The sectional view of the sternum with 30 mm elevation at the 3rd intercostal space.

When placing the Nuss bar in the 3rd intercostal space with the sternum elevated 15 mm, 20 mm 25 mm and 30 mm, respectively, as shown in Fig. 10, the Haller index in four cross sections changes by different degrees after the operation, of which, values are given in Tables 7~10. The operative plans of elevating 15 mm and 20 mm at the 3rd intercostal space cannot meet our demand while elevating 25 mm and 30 mm can. However, when the value of elevation is 30 mm, the sternum appears hypercorrected from the 2nd to 3rd intercostal space, as shown in Fig. 11. Thus, if placing the Nuss bar at the 3rd intercostal space, an elevation distance of 25 mm would be suitable.

**Table 7:** The Haller index of patient A after the operation by placing the Nuss bar at the 3rd intercostal space with the sternum elevated 15 mm.

Cross-section	B	A	The Haller index
a	86.329	235.63	2.729
b	89.076	240.64	2.702
c	89.826	247.72	2.758
d	83.246	250.48	3.009

**Table 8:** The Haller index of patient A after the operation by placing the Nuss bar at the 3rd intercostal space with the sternum elevated 20 mm.

Cross-section	B	A	The Haller index
a	91.072	231.23	2.539
b	94.098	236.64	2.515
c	94.848	243.72	2.570
d	88.268	247.98	2.809

**Table 9:** The Haller index of patient A after the operation by placing the Nuss bar at the 3rd intercostal space with the sternum elevated 25 mm.

Cross-section	B	A	The Haller index
a	95.760	227.83	2.379
b	99.120	231.72	2.338
c	99.870	239.98	2.403
d	93.290	244.38	2.620

**Table 10:** The Haller index of patient A after the operation by placing the Nuss bar at the 3rd intercostal space with the sternum elevated 30 mm.

Cross-section	B	A	The Haller index
a	100.508	224.58	2.234
b	104.142	228.14	2.191
c	104.892	237.32	2.263
d	98.312	242.13	2.463

**Figure 12:**
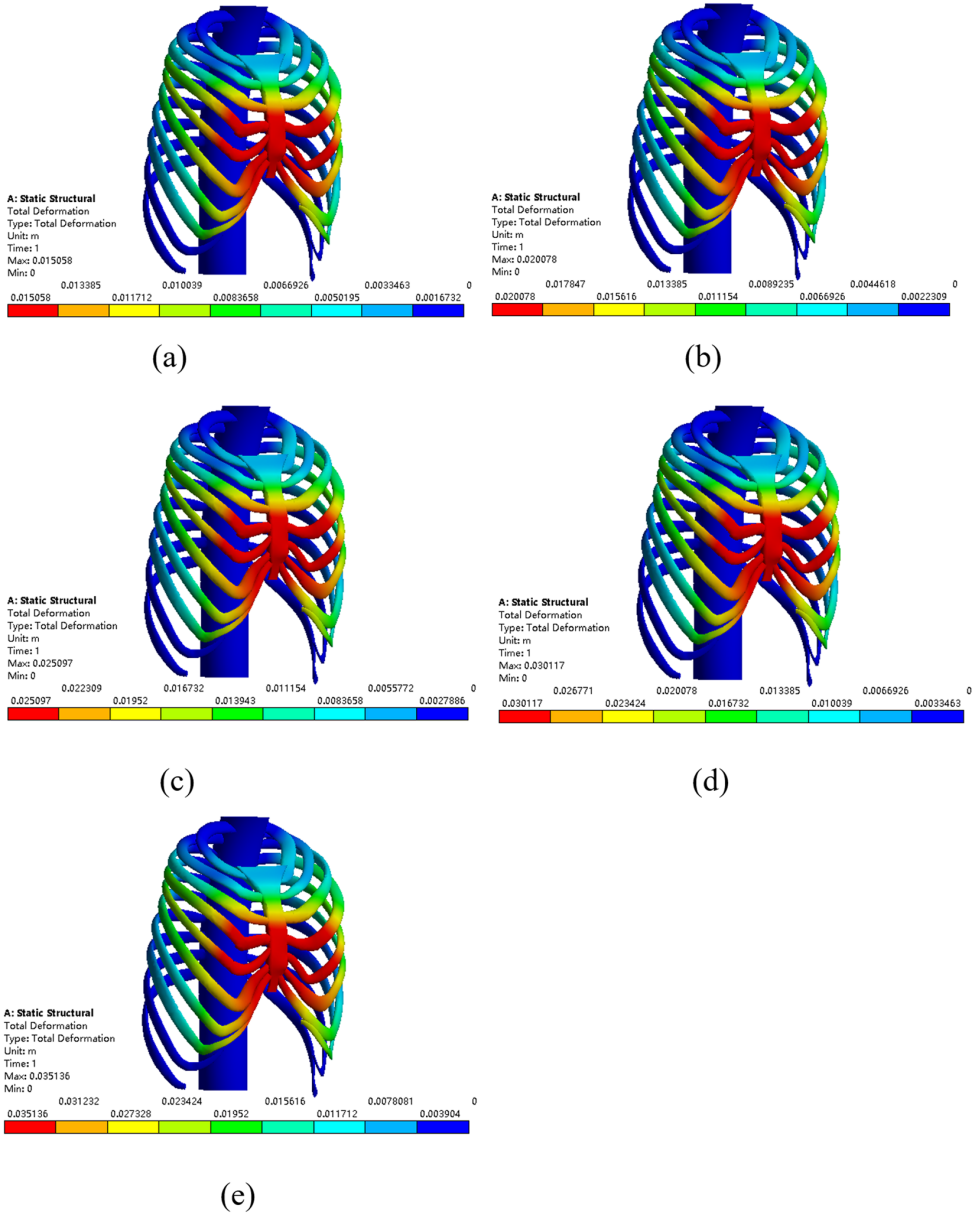
The correction effect of the chest model. (**a**) The sternum is elevated 15 mm at the 4th intercostal space. (**b**) The sternum is elevated 20 mm at the 4th intercostal space. (**c**) The sternum is elevated 25 mm at the 4th intercostal space. (**d**) The sternum is elevated 30 mm at the 4th intercostal space. (**e**) The sternum is elevated 35 mm at the 4th intercostal space.

**Figure 13:**
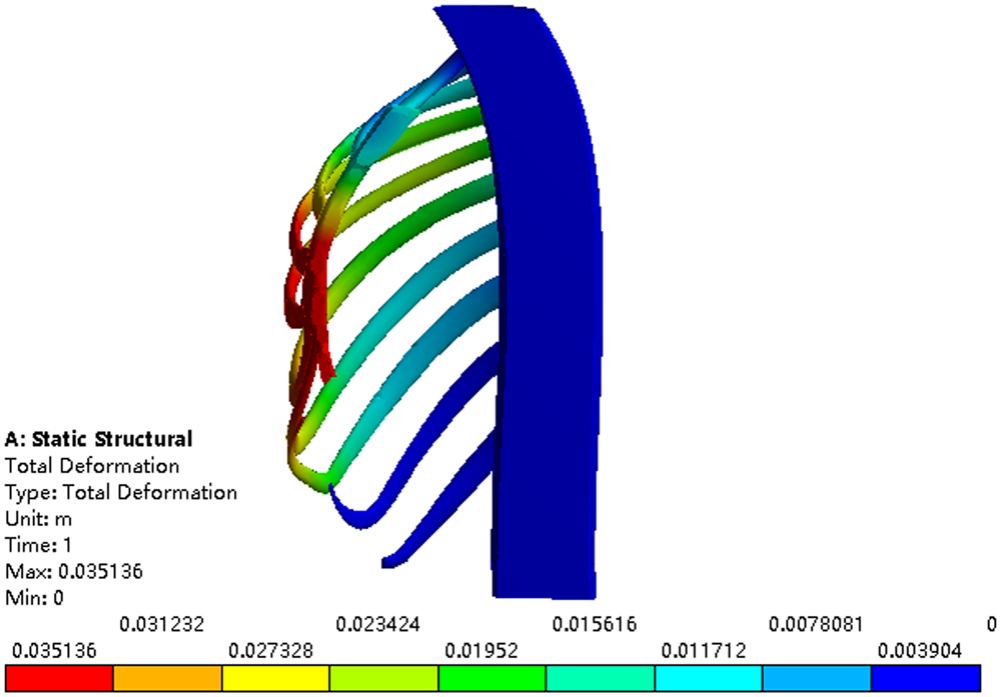
The sectional view of the sternum with 35 mm elevation in the 4th intercostal space.

**Figure 14:**
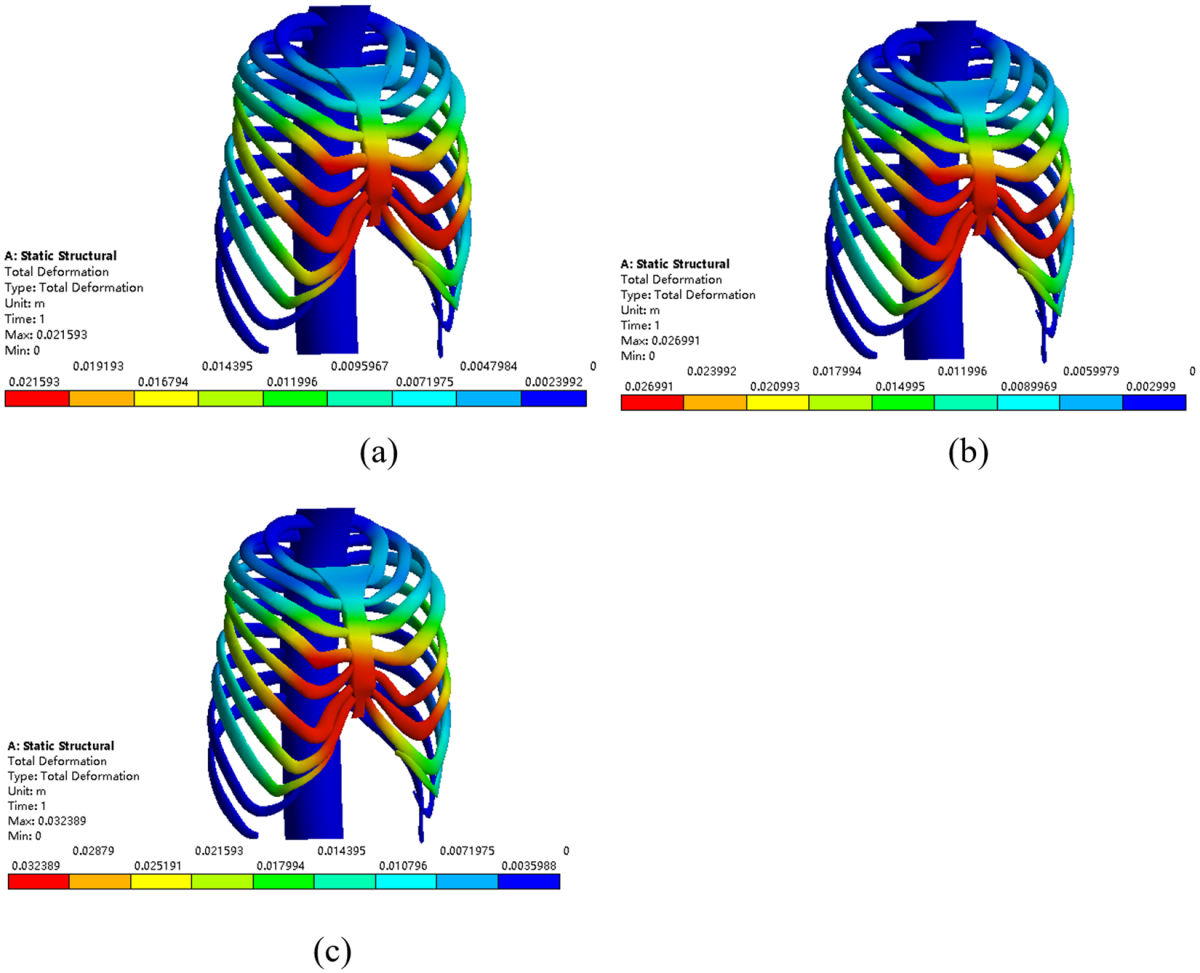
The correction effect of the chest model. (**a**) The sternum is elevated 20 mm at the 5th intercostal space. (**b**) The sternum is elevated 25 mm at the 5th intercostal space. (**c**) The sternum is elevated 30 mm at the 5th intercostal space.

**Figure 15:**
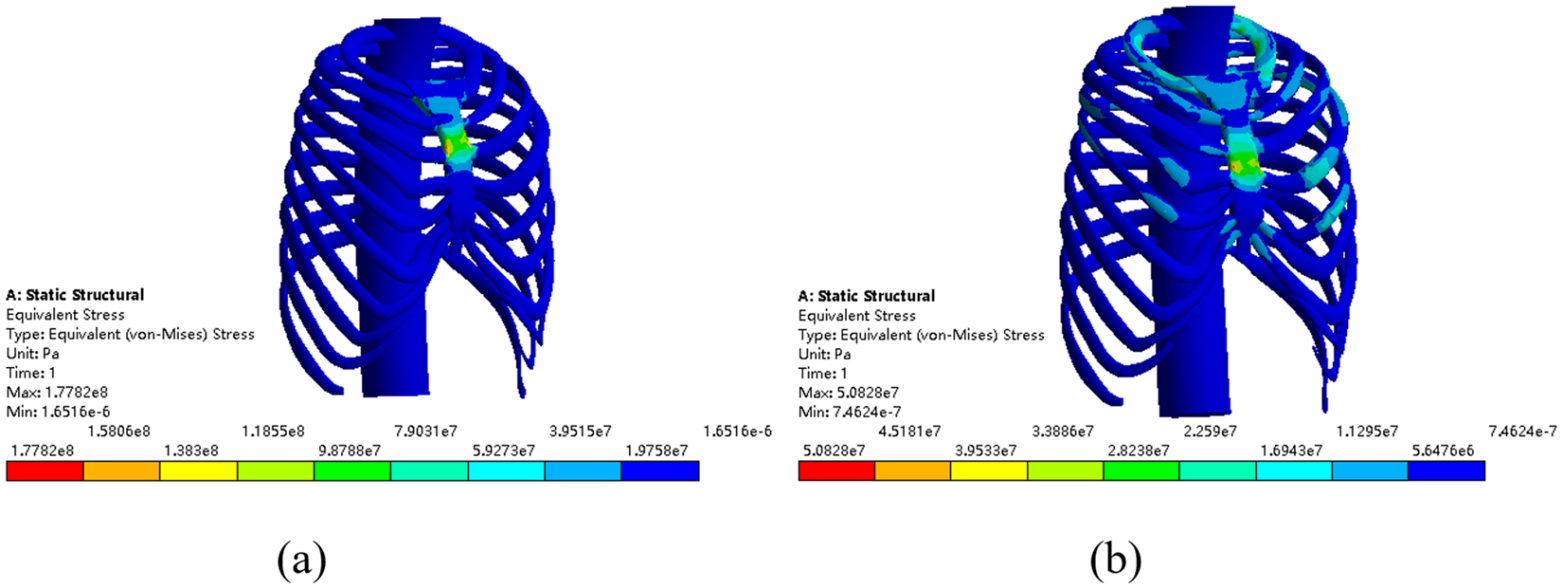
The equivalent stress of the model. (**a**) The sternum is elevated 25 mm in the 3rd intercostal space. (**b**) The sternum is elevated 30 mm in the 4th intercostal space.

## Discussion

**Figure 16:**
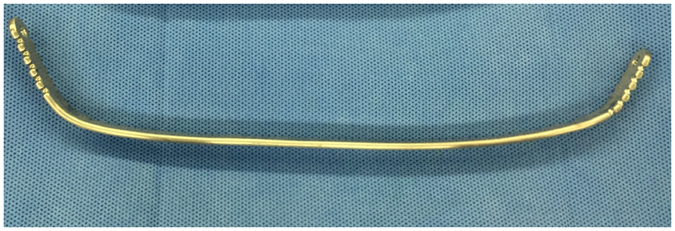
The prototype of the Nuss bar manufactured according to the CAD model.

**Figure 17:**
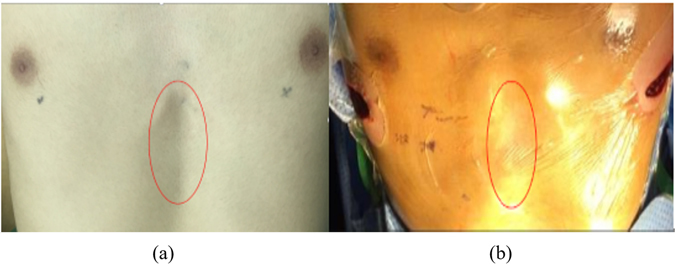
The thoracic shapes of pre- and post-operation. (**a**) The thoracic shape of the patient pre-operative. (**b**) The thoracic shape of the patient post-operative.

As discussed in the above sections, by using a three-dimensional finite element method, different operative plans are simulated and analyzed, and the optimal operative plan is determined according to both the chest wall configuration and the stress distribution. According to the CAD model of the Nuss bar, the prototype of the Nuss bar is manufactured, as shown in Fig. 16, to validate the Nuss bar design and its analysis. The Nuss bar was placed at the 4th intercostal space of the patient’s pectus excavatum chest. As shown in Fig. 17, comparing with the pre-operative chest (Fig. 17(a)), the thoracic deformity is corrected effectively, and the surgical result is also satisfactory to the patient. More than ten such PEX cases have been corrected using the method in this paper. From the practice of orthopedic operation of the funnel chest patient, the feasibility and rationality of our method is verified. Determining the shape and position of the Nuss bar before operation can shorten operation time, decrease blood loss and the possibility of damage to the patient, and ensure post-operative effects. In brief, this method is more accurate and individualized compared to conventional surgery.

The proposed approach in this study gave a new solution to design the Nuss bar and to determine the incision position in pectus excavatum surgery; the procedure of design and finite element analysis is conducted by hand and is employed several different software, including Mimics^®^, Solidworks^®^, Ansys^®^, and etc. These different software are independent and require different format, so that the transformation among these software platforms is inconvenient. So, it is time-consuming to deal with the design and analysis among these software platforms. The second limitation of this proposed approach is the manufacturing cost of customized Nuss bar, which is pretty higher than that of traditional method in Nuss operation. And thus, the future directions of this study will be focused on two aspects: (1) to develop an integrated software platform, which can be employed to reconstruct 3D model of skeleton from CT imaging data, to design the 3D model of Nuss bar, and to conduct finite element analysis; (2) to develop a quick and low-cost manufacturing method for customized Nuss bar, such as the 3D printing technology. With these two further researches, the design and manufacture of customized Nuss bar in pectus excavatum surgery can be greatly facilitated, and the proposed method in this study can be a good reference for other areas of surgery.

Similarly, placing the Nuss bar at the 4th intercostal space with the sternum elevated 15 mm, 20 mm 25 mm, 30 mm and 35 mm, respectively, as shown in Fig. 12, the Haller index in four cross sections were recorded and listed in Tables 11~14. The elevation distance of 15 mm and 20 mm at the 4th intercostal space cannot meet the requirement while an elevation distance of more than 25 mm can. However, when the elevation distance is over 30 mm, such as 35 mm, there appears to be hypercorrection in the sternum, as shown in Fig. 13. Compared to the effect of elevating 25 mm to that of 30 mm, the latter obviously has a better effect. Therefore, if we place the Nuss bar at the 4th intercostal space, an elevation distance of 30 mm would be suitable.

**Table 11:** The Haller index of patient A after the treatment by placing the Nuss bar at the 4th intercostal space with the sternum elevated 15 mm.

Cross-section	B	A	The Haller index
a	85.485	239.05	2.796
b	89.068	241.34	2.710
c	89.818	242.72	2.702
d	83.238	250.02	3.004

**Table 12:** The Haller index of patient A after the treatment by placing the Nuss bar at the 4th intercostal space with the sternum elevated 20 mm.

Cross-section	B	A	The Haller index
a	89.947	237.12	2.636
b	94.088	237.98	2.529
c	94.838	239.32	2.523
d	88.258	246.12	2.789

**Table 13:** The Haller index of patient A after the treatment by placing the Nuss bar at the 4th intercostal space with the sternum elevated 25 mm.

Cross-section	B	A	The Haller index
a	94.409	234.58	2.485
b	99.107	233.54	2.356
c	99.857	235.09	2.354
d	93.277	241.23	2.586

**Table 14:** The Haller index of patient A after the treatment by placing the Nuss bar at the 4th intercostal space with the sternum elevated 30 mm.

Cross-section	B	A	The Haller index
a	98.871	231.62	2.343
b	104.127	229.97	2.209
c	104.877	229.11	2.184
d	98.297	237.11	2.412

Finally, placing the Nuss bar at the 5th intercostal space with elevating the sternum 20 mm, 25 mm and 30 mm, respectively, as shown in Fig. 14, the Haller index in four cross sections are listed in Tables 15~17. All of these operative plans cannot meet the demand, and there appears to be hypercorrection in the sternum when elevated 30 mm or more. Therefore, it is not a suitable operative plan for patient A to place the Nuss bar at the 5th intercostal space.

**Table 15:** The Haller index of patient A after the treatment by placing the Nuss bar at the 5th intercostal space with the sternum elevated 20 mm.

Cross-section	B	A	The Haller index
a	86.495	242.86	2.808
b	95.603	242.07	2.666
c	96.353	241.83	2.510
d	89.773	244.92	2.728

**Table 16:** The Haller index of patient A after the treatment by placing the Nuss bar at the 5th intercostal space with the sternum elevated 25 mm.

Cross-section	B	A	The Haller index
a	90.094	239.16	2.655
b	95.005	237.81	2.503
c	101.751	238.23	2.341
d	95.171	240.25	2.524

**Table 17:** The Haller index of patient A after the treatment by placing the Nuss bar at the 5th intercostal space with the sternum elevated 30 mm.

Cross-section	B	A	The Haller index
a	93.693	239.16	2.553
b	99.201	232.81	2.347
c	107.149	234.97	2.193
d	100.569	235.02	2.337

Analysis indicates that placing the Nuss bar in the 2nd or 5th intercostal space is unable to meet the correction requirement. There are two suitable plans for patient A: placing the Nuss bar either at the 3rd intercostal space with 25 mm elevation or at the 4th intercostal space with 30 mm elevation. In addition to the evaluation of the Haller Index, the maximum stress of the chest after operation should also be taken into consideration. Comparing the maximum stress in the above-mentioned candidate operative plans, as shown in Fig. 15, the maximum stress of placing Nuss bar at the 4th intercostal space with 30 mm elevation is lower than the other, therefore, the operation plan is determined.

Based on the discussion above, the following conclusions can be drawn:

1. By using the CT imaging data and referring to a similar healthy person, the shape of the Nuss bar is designed for pectus excavatum patients.
2. It is found that the thoracic transverse of the chest wall boundary is almost convex, the Nuss bar shape is finely tuned to be convex to ensure the orthopedic effect.
3. By using the CT imaging data of a PEX chest, the thoracic structure is reconstructed for finite element analysis. Through the analysis of different operation plans of incision position and sternum elevation, the optimal operation plan is determined by evaluating the Haller index and the stress distribution after the Nuss bar implantation.
4. Experiments were conducted to validate the method in this paper. Determining the shape and position of the Nuss bar before operation can shorten the operation time, decrease blood loss and the possibility of damage to the patient, and ensure post-operative effects.
